# Editorial: Big Data and machine learning in cancer theranostics

**DOI:** 10.3389/fdata.2022.972726

**Published:** 2022-07-19

**Authors:** Fabricio Alves Barbosa da Silva, Jack Adam Tuszynski, Helder Nakaya, Channing J. Paller

**Affiliations:** ^1^Scientific Computing Program, Oswaldo Cruz Foundation (Fiocruz), Rio de Janeiro, Brazil; ^2^Department of Physics, University of Alberta, Edmonton, AB, Canada; ^3^Hospital Israelita Albert Einstein, São Paulo, Brazil; ^4^The Johns Hopkins Hospital, Johns Hopkins Medicine, Baltimore, MD, United States

**Keywords:** Big Data, machine learning, cancer, systems biology, computational biology

Cancer is a growing global health problem. The World Health Organization (WHO) predicts 27 million new cancer cases worldwide by 2030. Cancer treatment includes several standard protocols that may cause harmful side effects. Nevertheless, after two decades of genomics technological breakthroughs, personalized medicine is being used to improve treatment outcomes and mitigate side effects.

Machine learning is a consolidated methodology for extracting knowledge from omics Big Data. Essentially, machine learning algorithms on biology reveal underlying patterns, design models, and make statistical predictions using omics data as training datasets. We currently see the application of machine learning algorithms in several domains, such as genomics, proteomics, and systems biology. For example, machine learning algorithms for cancer research have been used to identify potential therapeutic targets, propose adjuvant therapies, and define measures to minimize side effects.

The pace of growth of omics data in recent years is astonishing. This speedy growth of omics data volume is particularly true for single-cell RNA-seq datasets. Therefore, innovative machine learning strategies using Big Data are needed to generate new knowledge from ever-growing amounts of cancer Big Data. Furthermore, it is of utmost importance to deploy high-performance and high-throughput computing platforms and tools to achieve this objective accurately and efficiently. Therefore, we proposed the Research Topic “*Big Data and Machine Learning in Cancer Theranostics*” to gather contributions describing the current state of the art in Big Data and Machine Learning methods in cancer theranostics. One main focus is on analyzing large omics datasets on scalable computational infrastructures.

Four papers were accepted for publication in this Research Topic from 11 submissions. The evaluation process was highly selective, and we acknowledge all reviewers that participated in that process. Below we present a summary of the four papers included in this Research Topic.

The original research paper “*Data-Driven Modeling of Breast Cancer Tumors Using Boolean Networks*” by Sgariglia et al. proposed a methodology for building data-driven boolean networks that model the dynamics of breast cancer tumors. The authors defined network components and structure based on RNA-seq data of breast cancer cell lines. They used a Boolean logic formalism to describe the network dynamics by identifying basins of attraction of the epigenetic landscape related to cancer cells. Single-cell RNA-seq data from breast cancer tumors and interactome information enabled the study of the dynamics of malignant subnetworks of up-regulated genes by identifying basins of attractions related to tumor cells on a personalized medicine approach.

The opinion article “*Towards Machine-Readable (Meta) Data and the FAIR Value for Artificial Intelligence Exploration of COVID-19 and Cancer Research Data*” by Campos et al. debates how the explosion of omics Big Data has considerably changed the landscape of cancer research. In recent years, heterogeneous data integration has been more common in dealing with complex biological problems, including cancer. According to the authors, it is a consensus that individual research centers cannot produce enough data to generate accurate prognostic and predictive models. Therefore, data integration in precision oncology is a mandatory step to generate novel results. Finally, the authors discuss how adopting the FAIR (Findable, Accessible, Interoperable, and Reusable) data principles in cancer research impacted data management in other areas, such as COVID-19 research.

In the review article “*A Survey on Human Cancer Categorization Based on Deep Learning*” by Ibrahim et al., the authors assess deep learning concepts related to medical imaging research and survey several contributions in the field. In addition, it covers the main categories of imaging procedures related to cancer. The survey also comprises the usage of deep learning for cancer diagnosis, detection, and prognosis. Furthermore, the authors discuss challenges and trends for upcoming research.

In the opinion article “*Digital Slide Assessment for Programmed Death-Ligand 1 Combined Positive Score in Head and Neck Squamous Carcinoma: Focus on Validation and Vision*,” by Eccher et al., the authors discuss how the introduction of immunotherapy drugs targeting the interaction between programmed death-1 protein (PD-1) expressed on T-helper lymphocytes, and the programmed death-ligand-1 (PD-L1) expressed on cancer cells represented a turning point in the therapy of several aggressive cancers in an advanced stage, including Head and Neck Squamous Carcinoma (HNSCC). The authors also anticipate deploying Artificial Intelligence tools in clinical practice to assess PD-L1 Combined Positive Score in HNSCC and select suitable immunotherapy patients.

## Author contributions

All authors listed have made a substantial, direct, and intellectual contribution to the work and approved it for publication.

## Conflict of interest

The authors declare that the research was conducted in the absence of any commercial or financial relationships that could be construed as a potential conflict of interest.

## Publisher's note

All claims expressed in this article are solely those of the authors and do not necessarily represent those of their affiliated organizations, or those of the publisher, the editors and the reviewers. Any product that may be evaluated in this article, or claim that may be made by its manufacturer, is not guaranteed or endorsed by the publisher.

